# Hospital Admission and Discharge: Lessons Learned from a Large Programme in Southwest Germany

**DOI:** 10.5334/ijic.6534

**Published:** 2023-01-27

**Authors:** Johanna Forstner, Maximilian Pilz, Cornelia Straßner, Aline Weis, Nicola Litke, Lorenz Uhlmann, Frank Peters-Klimm, Frank Aluttis, Annika Baldauf, Marion Kiel, Markus Qreini, Petra Kaufmann-Kolle, Janina Schubert-Haack, Nadja El-Kurd, Katrin Tomaschko-Ubeländer, Sarah Treffert, Ronja Rück, Bärbel Handlos, Gökce Karakas, Michel Wensing, Joachim Szecsenyi

**Affiliations:** 1 Department of General Practice and Health Services Research, University Hospital Heidelberg, Im Neuenheimer Feld 130.3, Marsilius Arkaden, Turm West, 69120 Heidelberg, DE; 2 Institute of Medical Biometry, University Hospital Heidelberg, Im Neuenheimer Feld 130.3, Marsilius Arkaden, Turm West, 69120 Heidelberg, DE; 3 aQua-Institute, Maschmühlenweg 8–10, 37073 Göttingen, DE; 4 AOK Baden-Württemberg, Presselstraße19, 70191 Stuttgart, DE; 5 HÄVG Hausärztliche Vertragsgemeinschaft Aktiengesellschaft Regionaldirektion Süd, Kölner Str. 18, 70376 Stuttgart, DE; 6 Gesundheitstreffpunkt Mannheim, Max-Joseph-Str. 1, 68167 Mannheim, DE

**Keywords:** care transition, admission management, post-discharge provider follow-up, patient readmission, continuity of patient care, strong primary care, integrated care

## Abstract

**Introduction:**

In the context of a GP-based care programme, we implemented an admission, discharge and follow-up programme.

**Description:**

The VESPEERA programme consists of three sets of components: pre-admission interventions, in-hospital interventions and post-discharge interventions. It was aimed at all patients with a hospital stay participating in the GP-based care programme and was implemented in 7 hospitals and 72 general practices in southwest Germany using a range of strategies. Its’ effectiveness was evaluated using readmissions within 90 days after discharge as primary outcome. Questionnaires with staff were used to explore the implementation process.

**Discussion:**

A statistically significant effect was not found, but the effect size was similar to other interventions. Intervention fidelity was low and contextual factors affecting the implementation, amongst others, were available resources, external requirements such as legal regulations and networking between care providers. Lessons learned were derived that can aid to inform future political or scientific initiatives.

**Conclusion:**

Structured information transfer at hospital admission and discharge makes sense but the added value in the context of a GP-based programme seems modest. Primary care teams should be involved in pre- and post-hospital care.

## Abstract (de)

**Einleitung:**

Im Rahmen der hausarztzentrierten Versorgung wurde ein Programm zur Verbesserung von Krankenhausaufnahmen und -entlassungen sowie der Nachsorge implementiert.

**Beschreibung:**

Das VESPEERA-Programm bestand aus verschiedenen Komponenten, die vor der Aufnahme, während des Krankenhausaufenthalts sowie nach der Entlassung durchgeführt wurden. Das Programm richtete sich an alle Patienten mit einem Krankenhausaufenthalt, die an der hausarztzentrierten Versorgung teilnahmen und wurde in 7 Krankenhäusern und 72 Hausarztpraxen in Südwestdeutschland unter Anwendung einer Reihe von Strategien implementiert. Seine Wirksamkeit wurde anhand des primären Endpunkts “Rehospitalisierungen innerhalb von 90 Tagen nach der Entlassung” bewertet. Anhand einer Fragebogenbefragung beim Personal wurde der Implementierungsprozess untersucht.

**Diskussion:**

Ein statistisch signifikanter Effekt konnte nicht gefunden werden, die Effektgröße war jedoch ähnlich wie bei anderen Interventionen. Die Interventionstreue war gering. Kontextfaktoren, die die Implementierung beeinflussten, waren unter anderem verfügbare Ressourcen, externe Anforderungen wie gesetzliche Vorschriften und die Vernetzung von Leistungserbringern. Es wurden Schlussfolgerungen gezogen, die für künftige politische oder wissenschaftliche Initiativen hilfreich sein können.

**Schlussfolgerung:**

Eine strukturierte Informationsweitergabe bei der Aufnahme und Entlassung aus dem Krankenhaus ist sinnvoll, doch scheint der zusätzliche Nutzen im Rahmen der hausarztzentrierten Versorgung begrenzt. Teams der Primärversorgung sollten in die prä- und poststationäre Versorgung einbezogen werden.

## Background

Hospital discharges are critical moments regarding continuity of care. They can have negative impacts on quality of care and outcomes of patients [1,2,3], providers’ satisfaction [3] and health system efficiency [4]. Improving care transitions and reducing hospital readmissions is of relevance in many health systems. There is a range of interventions that have shown benefits, especially those consisting of multiple pre- and post-discharge components. The majority of these interventions are hospital-based and mostly provided around or after hospital discharge [5,6,7,8,9,10,11].

Strong primary care, including a central role in hospital admission and discharge [12], improves care coordination, reduces hospital admissions in ambulatory care sensitive conditions and hospital readmissions [13,14,15,16]. Nevertheless, few studies that involved primary care in interventions after hospital discharge showed measurable positive effects. For example, Brooke et al. [17] found that early primary care follow-up after hospital discharge significantly reduced the number of readmissions within 30 days in patients after high-risk surgery. In a study conducted by Balaban et al. [18], patients who received follow-up care by a primary care provider had fewer undesirable outcomes. An umbrella review conducted by Straßner et al. [6] showed that admission management was considered to be crucial but that it was lacking in all of the studies included. Therefore, the authors recommend including admission management in future studies. Lee et al. [19] demand to ‘focus on providing transitional care within the entire cycle of care […] from time of admission to final transition to the primary care setting’ [19](p. 8).

In southwest Germany, a programme to develop strong primary care was initiated more than a decade ago (general practitioner (GP)-based care programme) [16]. A logical next step was to include primary care in care transitions and readmission prevention programmes including standardised care pathways between primary care and hospital care. Thus, vertical integration of care can be improved [20]. Consequently, the VESPEERA programme addressed at adults with all-cause hospitalisation was developed as an add-on to the GP-based care programme. The VESPEERA programme is a complex multi-component transitional care intervention that was implemented in a complex context (such as different types of organisations). Therefore, the effectiveness evaluation was accompanied by an extensive process evaluation. In this paper we present insights into the effects of the VESPEERA programme, experiences with its’ implementation as well as the lessons learned during implementation and evaluation.

## Description of the care practice

The VESPEERA programme was developed based on the following several pillars: It was informed by a review of the international research evidence [6] as well as a review of quality deficits and potential for improvement in Germany [21]. Additionally, the programme was aligned with a legal regulation obligating hospitals to implement comprehensive measures to improve discharge management, which came into place in October 2017 (*Rahmenvertrag Entlassmanagement*) [22]. This regulation demands to improve and intensify information flow and communication between hospitals and other care providers, to identify patients with complex care needs, to consider information about the patients’ situation before hospital admission and to inform patients about measures taken regarding their discharge. Furthermore, the programme was aligned with the GP-based care programme, which places general practitioners in a coordinating role. The experiences and requirements by the stakeholders were considered by involving them in the development process in the form of workshops in which they discussed the intervention components (see also ‘Implementation strategies’). In the following, the intervention, its implementation, the methodological approach and the results of the evaluation will be presented.

### Context, in which the care practice was implemented

Hospital care and ambulatory care in Germany have traditionally been strongly separated and insufficiently coordinated. Typically, hospitals should only be accessed when the possibilities of the outpatient sector are not sufficient for meeting patients’ care needs. In this case, ambulatory physicians (GPs or other specialists) admit patients to a hospital, which the patient can choose freely [23]. The admitting physician is encouraged to provide the hospital with all relevant information about the patient’s medical history as well as diagnostics and therapy that were already applied prior to admission [24]. However, the amount and quality of information differ. Even though technically, access to hospitals is restricted, many patients enter the hospital through the emergency department and without the involvement of any ambulatory physicians [23]. During the hospital stay, contact between hospital staff and ambulatory physicians is rare [12]. At discharge, discharge letters are mandatory [24] but often arrive late and with missing information. Personal contact, i.e. via telephone, is not required and thus depends on individual motivation [25]. The legal regulation mentioned above aims at standardising and improving hospital discharges (*Rahmenvertrag Entlassmanagement, § 39 Abs. 1a, Social Code Book V)*. Primary care is predominantly provided by physicians, nurses are rarely involved in primary care in Germany. Physicians are supported by medical assistants, who mostly have administrative and simple medical tasks [26]. In 2008, the concept of the care assistant in general practice (VERAH, *Versorgungsassistentin in der Hausarztpraxis*) was introduced with the aim to reduce physician burden and to take over more comprehensive tasks. After having absolved an additional training, VERAHs can take over tasks such as case management, coordination of care, routine home visits etc. [27].

### Description of intervention components

**Figure 1:**
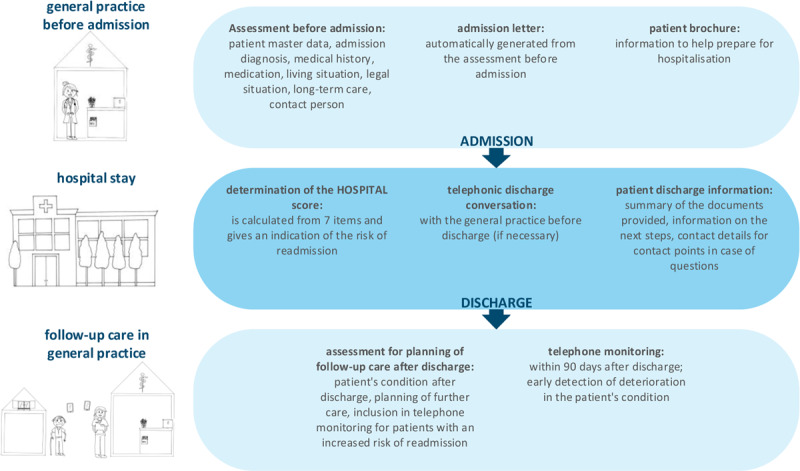
Components of the VESPEERA programme, figure by Forstner et al. [29] licensed under CC BY 4.0.

The VESPEERA programme consists of a set of components that were applied depending on the type of hospital admission and time point of study enrolment (see Figure 1). The three sets of components were: pre-admission interventions, in-hospital interventions and post-discharge interventions.

Pre-admission interventions were delivered in the general practice, mainly by the VERAH under the responsibility of the GP and in a separate room. They include a structured and standardised computer-aided **assessment before admission** resulting in an **admission letter**. The information on the reason for hospital admission, the patient’s medical history, medication, living situation, long-term care situation including the availability of medical aids and appliances as well as the patient’s legal situation were collected by the VERAH in a designated additional software (“CareCockpit”) and automatically included in the admission letter. Furthermore, the letter included contact and reachability information of the general practice. The time of application was not pre-defined and depended on the urgency of the admission but recommended to be as close to the admission as possible to ensure that the information was up to date. Furthermore, the patients received a paper-based **patient brochure** that aids in preparing for a hospital stay with information on relevant documents and items to bring, patients’ rights and obligations as well as information on contact points for further support. The brochure was written in simple language and complemented by pictograms.

Hospitals were responsible for integrating the VESPEERA admission letter into their processes in a way that it was accessible by all health professionals involved. Other in-hospital interventions were performed around discharge: In cases where needed, a **telephonic discharge conversation** of the hospital staff with the GP was performed. No sharp criteria were defined for cases where this conversation might have been necessary but a list was provided as orientation (one-page pdf-file, provision to staff by hospital management according to internal processes). The decision on whether a physician, a nurse or another health professional was responsible for the discharge conversation of individual patients was made by the hospitals. The **HOSPITAL score** [28] was to be computed before discharge and shared with the general practice via the discharge letter. The HOSPITAL score consists of seven items (low haemoglobin, discharge from oncological services, low sodium, procedures, emergency admission, number of hospital admissions in the preceding year, and length of stay) and can help to identify patients with an increased risk for readmission. Patients received a paper-based **patient discharge information** in simple language, providing an overview of documents, next appointments with the GP and the hospital, as well as contact information of the hospital and self-help groups. If hospitals had already implemented a similar document, it was not necessary to provide the patient with the VESPEERA discharge information but adaption to its contents was recommended.

Post-discharge interventions in the general practice were also conducted in the CareCockpit software by the VERAH and include a structured and standardised **assessment for planning of follow-up care after discharge**. It includes medical, social, long-term needs such as wounds, pain, medication, involvement of other health professionals and the option to compute the HOSPITAL score in case it was not provided by the hospital. It was recommended to perform this assessment timely after discharge, if possible, on the next working day. The patient received a brief paper-based summary of the arranged care plan which could be printed from the software. Patients with an increased risk for readmission (HOSPITAL score ≥ 5) were included in a structured and standardised three-month **telephone monitoring** which included a monitoring of symptoms as well as of the arranged care plan. If patients were in the practice regularly, the monitoring could be combined with in-practice visits and did not need to be additional telephonic appointments. In case of need, paper-based sheets could be printed from the software to bring these to a home visit. The telephone monitoring included two mandatory appointments: one within a time frame of two weeks after discharge and one closing appointment three months after discharge. The number and frequency of in-between appointments was to be determined by the GP, based on their appraisal of the patients’ needs and adherence, the medical urgency and other possibly relevant factors.

### Implementation strategies

Several strategies were applied to implement the intervention in general practices and hospitals. First, representatives of hospitals, general practices, health insurers and patients were involved in the development of the intervention to increase the acceptance of the programme. All components and their items were discussed in detail regarding their relevance, wording, design etc. Second, hospitals were able to adapt the delivery of the intervention according to organisational resources. They could independently decide whether they work paper-based or electronically or which kind of health profession they give responsibility for implementing the intervention components. A description of the individual implementation was to be provided to the study team. Third, the intervention components in general practices were delivered using the CareCockpit software which is a self-developed case management software. Its’ previous version, the PraCMan-Cockpit, is widely used in southwest Germany [28] and has been enhanced by integrating the VESPEERA assessments. The assessments within the CareCockpit software are standardised and questions are phrased in a way so that they could directly be asked to patients. Fourth, GPs and VERAHs were trained in the handling of the software in a role-play format and the study related procedures in a 2.5h training using a train-the-trainer strategy. The training mostly focused on the contents of the assessments, function of the software and requirements regarding the study design. Fifth, hospitals and general practices were provided with various different educational materials (such as flowcharts or video tutorials) and handling guidelines. Sixth, in addition to the educational materials, the whole study team provided ongoing consultation by telephone and by site visit, if necessary. This included support with study-related procedures such as obtaining informed consent, checking the status of and support with implementation or IT-support with the CareCockpit software. Seventh, feedback was provided to hospitals and general practices in the form of annual feedback reports and two feedback meetings within three years. The feedback reports included individualised results of the evaluation to point out potential for improvement. The feedback meetings gave an opportunity to bring together care providers from hospitals and general practices and offered an opportunity to exchange ideas, experiences and perspectives. Finally, several financial incentives such as fee-for-service financing for the provision of health care services and lump sums for study participation for hospitals were offered. A more detailed description of the implementation strategies and their intention can be found in the study protocol of the process evaluation [30].

### Evaluation design and methods

#### Study design, participants and setting

The VESPEERA programme was implemented in seven hospitals and 72 general practices in nine pre-defined districts in southwest Germany. It was expected that 7,088 patients resulting in 11,340 hospital admissions would participate in the multi-centre controlled study, which was conducted between May 2018 and September 2019. Inclusion criteria were admission to/discharge from hospital, age 18 years and older and participation in the GP-based care programme of the health insurer AOK Baden-Wuerttemberg (this implies that the GP provides comprehensive healthcare and coordinates hospital access [16]). Patients residing in nursing homes were excluded from study participation.

The effectiveness study was accompanied by a structured survey among care providers from participating general practices and hospital departments to explore the implementation processes between November 2019 and April 2020.

#### Outcomes and data sources

The effectiveness of the VESPEERA programme was measured by the primary outcome ‘number of readmissions due to the same indication within a time frame of 90 days’ (same indication was defined as the same three-digit ICD-10-GM code as the main diagnosis at discharge) as well as a range of secondary outcomes such as the number of admissions due to ambulatory care-sensitive diagnoses, delayed prescriptions of medications or medical aids and appliances and referrals to rehabilitation therapeutics on a case level. The analysis was conducted using a data set consisting of claims data (so called secondary data according to *Social Code Book V*) and data collected within the CareCockpit (so called primary data).

Questionnaires used in the quantitative survey were self-developed as paper-based questionnaires and based on preceding qualitative interviews with care providers involved in the VESPEERA programme [29]. They included statements on the working mechanism of the programme, acceptance of the individual intervention components, various contextual factors, perceived outcomes, attractiveness and acceptance of the intervention and sociodemographic information. Five-point Likert scales were used to indicate whether agreement by the participants with the statements was ‘not at all true’ up to ‘very true’.

#### Data analysis

The endpoints were analysed using difference-in-difference models [31]. The change of the primary outcome (six months before vs. three months after the intervention) of the intervention group was compared to the change in the control group, which was built from claims data using propensity score matching. As the outcomes are binary and data has a hierarchical structure, random and fixed effects were considered resulting in a mixed logistic regression model. As a sensitivity analysis, the primary endpoint was evaluated using interrupted time series models that take time trends into account [32]. Furthermore, several subgroups were analysed in order to identify populations with high or low effectiveness of the intervention conducting the primary analyses in those groups.

A fidelity score was created using the respondents’ replies to whether they have used each of the intervention component at least once or are familiar with its content (the latter applies to brochures that are handed out to patients). The maximum number of components to be used was set to 1 (=100%), the result is a score between 0 and 1 indicating the degree of fidelity.

#### Ethical considerations

The effectiveness evaluation was registered (DRKS00014294 on DRKS/Universal Trial Number (UTN): U1111-1210-9657) and approved by the ethics committee of the Medical Faculty Heidelberg prior to the start of the study (S-071/2018), as well as the ethics committee of the State Chamber of Physicians of Baden-Württemberg (B-F-2018-023). The process evaluation was registered (DRKS00015183 on DRKS/Universal Trial Number (UTN: U1111-1218-0992) and approved by the ethics committee of the Medical Faculty Heidelberg prior to the start of the study (S-352/2018). Data linkage for the data set for the effectiveness evaluation was carried out by an independent institute using pseudonymised patient IDs. After data linkage and before the transfer to the evaluating institution, IDs were pseudonymised once again. All patients and care providers participating in the study gave their informed written consent.

#### Methodological remarks

The TIDieR checklist was used for the description of the intervention [33]. There is one deviation to the methods of the effectiveness evaluation as published in the study protocol. Originally, for the primary analysis, study arm 1 (patients with a planned admission in a participating hospital) was to be compared to the control group. However, no patient received in-hospital interventions, which renders study arm 2 (patients with a planned admission in a non-participating hospital) obsolete. Furthermore, the overall sample size was much lower than expected. Consequently, we decided to combine all study arms for the analysis of the intervention vs. the control group to increase the chance of detecting the underlying effect of the intervention.

Detailed information on the methods of the effectiveness and the process evaluation can be found in the respective study protocols [30,34].

### Results

#### Results of the effectiveness evaluation

During the intervention phase, 371 patients who fulfilled the inclusion criteria were admitted in 986 hospital admission cases. In total, 742 patients with 1,971 cases were considered in the analysis.

In total, the patients were between 18 and 99 years and on average 70 years (standard deviation (SD) 16) old. Male and female gender was equally represented. Patients in the intervention group were admitted on average 2.7 times (SD 2.3). The average length of stay was 7.4 days (SD 9.1) with a range of 0 to 69 days in the intervention group and 0 to 135 days in the control group. Using the classification of Huang et al. [35], patients had moderate comorbidity. The top five main diagnoses of the overall study population as well as those of patients with readmissions were mostly related to diseases of the heart and the lung. An overview of the characteristics of patients included in the study participants can be found in additional file 1.

Regarding the primary outcome (readmissions within 90 days due to the same indication), the rate after the intervention period was almost the same in both groups. In the control group, the readmission rate increased by 3.5% from 9.5% to 13%. In the intervention group, a decrease by 2.5% from 15.8% to 13.3% was observed. Altogether, a difference of 6% regarding readmission rates between intervention and control group was thus observed. Additional file 2 provides an overview of the descriptive results of the primary and secondary outcomes. The primary analysis did not show a significant effect (p = 0.385), although the intervention patients showed a slightly better outcome (odds ratio (OR) = 0.662). No significance tests were performed for any secondary outcomes. Additional file 3 provides an overview of the results of the statistical analysis. The sensitivity analysis confirmed the results of the primary analysis. Subgroup analyses showed that in most subgroups the OR was in favour of the intervention group. Regarding patients with severe comorbidity (high Charlson Comorbidity Index (CCI)), the likelihood for readmission was remarkably lower in the intervention group than in the control group (OR = 0.113, see additional file 4).

#### Results of the survey with care providers

The majority of care providers who participated in the quantitative survey were from general practices and, on average, the participants had 23 years of professional experience. Experience with the VESPEERA programme differed, participants on average had taken care of 9.1 patients (SD 21, see additional file 5).

Most of the intervention components were used at least once/known by about half of participants. Individual fidelity according to self-reports is 0.4, i.e. 40% on average, with quite high variation (see additional file 6).

The participants are rather indecisive when asked to rate the benefit over the expenses to use the intervention components with a tendency to a positive balance (means between 3.2 and 3.7). Regarding the assessment before admission and the admission letter, no clear recommendation for its utilisation is given by the participants. An additionally conducted linear regression model showed that the factor ‘nstitution’ was the only significant influencing factor, participants from hospitals rated its benefit higher than participants from general practices. As to the working mechanism of these intervention components, the participants think that they partially contribute to obtaining relevant information about the patient, especially social information relevant for social services. The participants do not think that admission processes in the clinic are accelerated by an admission letter. Approx. half of the participants believe that the benefits of the assessment/the admission letter are worth the effort. The assessment for planning of follow-up care after discharge is evaluated positively by the respondents from general practices. They agree that it is a suitable instrument to plan the patient’s care after discharge in a structured and complete way. Despite the positive evaluation, the effort to conduct the assessment is seen as quite high compared to the benefit. Regarding the telephone monitoring, participants from general practices see it as a suitable tool to check adherence to therapy and to identify further needs for patients with rather complex health care needs. Furthermore, they think that it can contribute to help to avoid rehospitalizations. More than half of the participants believe that the benefits of the telephone monitoring exceeds the effort. More results on the working mechanism of the intervention components can be found in additional file 7.

Several aspects regarding contextual factors have been addressed in the questionnaires. Concerning networking aspects, most of the participants stated that they have been working with the care providers in their region for many years. However, the utilisation of networking opportunities as well as personal contacts between care providers vary greatly. In general, the resources available for implementing the VESPEERA programme and admission and discharge management in general is described as insufficient by the majority of participants. Furthermore, many participants agree that external requirements such as legal regulations concerning data protection hinder cross-sectoral care (see additional file 8).

Almost half of the participants agreed that their awareness of the importance of cross-sectoral cooperation increased as a result of the VESPEERA programme. However, agreement with statements to improve cross-sectoral cooperation as a result of the VESPEERA programme (closer contact, new contacts, better provision of information and in general) are low (see additional file 9). On the other hand, there is a tendency for participants to wish for more comprehensive implementation of the VESPEERA programme, such as implementing it in all hospitals. Participants partially agree that the VESPEERA programme strengthens the role of the GP and the VERAH. Although implementation can be delegated to some extent, the majority of participants see the implementation as unwieldy (too bureaucratic, associated with double documentation and difficult to integrate into internal processes, see additional file 10).

## Discussion

The aim of this study was to examine the effects of an admission, discharge and follow-up intervention consisting of several components in hospitals and general practices and factors determining its implementation. A statistically significant effect of the intervention on patients’ hospital readmission rates could not be found. However, the results of the statistical analysis showed trends that patients might have benefitted from the intervention. For most outcomes, the odds ratios are in favour of the intervention group. Intervention fidelity was low and contextual factors that affected the implementation of the intervention are available resources, external requirements such as legal regulations, networking between care providers and belief in its working mechanism.

### Evaluation of the effectiveness of the VESPEERA programme

There are various explanations for the absence of statistically significant effects. We can observe lower rates of readmission than assumed and low rates compared to studies looking at similar populations [15]. Furthermore, patients who participate in the GP-based care programme show lower readmission rates than patients outside of the programme [15] to start with, therefore, we can expect a potential overlay of effects of the GP-based care programme and the intervention. Together with an overall small sample size and a heterogeneous and overall low intervention fidelity, this is the most probable explanation for the absence of effects. The low sample size also required adjustments of the planned evaluation: as we did not achieve statistical power, we had to merge the different study arms into one. Therefore, and even though this is a common difficulty in the evaluation of multicomponent interventions [6], we were not able to detect the contribution of the intervention components to potential effects. The low sample size can partly be explained by a lower number of participating general practices than expected, misunderstandings regarding study participation (e.g. practices thought that they could only include patients if they were admitted to one of the participating hospitals, or thought that patients could only be included before hospital admission), pre-selection of patients by general practices presumably leads to a selection bias (which we also could not control for by the propensity score matching) and problems regarding implementation. The comprehensiveness of the intervention components hindered patient study inclusion and data collection. Our evaluation benefited from using claims data. Even though insurance claims data are associated with limitations, they probably provide valid and comprehensive data on hospital admissions and they do not induce attention bias (or Hawthorne effect) as other types of data-collection might. Relying on claims data allowed us to analyse readmissions within 90 days without a recall bias, a time frame not typical in evaluations of interventions to reduce readmissions [36].

Nevertheless, the decrease in readmission rates in the intervention group and the increase in the control group (which corresponds to the overall trend in this population [15]) adds up to an effect of 6%. Compared to a meta-analysis on the effect of continuity of care interventions on readmissions between 30 and 90 days after discharge in elderly patients with chronic conditions [36], the reported risk ratio of 0.74 (95% CI, 0.65–0.84, p < 0.001) translates into an odds ratio of 0.82, which is similar to our result of an effect size of 6% with an odds ratio of 0.66. We therefore assume that there is an effect of our intervention on readmissions within 90 days after discharge and that we would have been able to show its significance within a larger study population.

### Implementation of the VESPEERA programme

The process evaluation provided insights into the working mechanisms of the intervention components, acceptance of the intervention and intervention fidelity and offers explanations for the small sample size. It showed that there were context-related barriers to the implementation of the VESPEERA programme such as limited resources in the organisation (e.g. staff, working places) or external regulations (e.g. data protection). Especially, the participation rate on the side of hospitals was low. They were occupied with the implementation of the legal regulation to improve discharge management (*Rahmenvertrag Entlassmanagement*) running parallel in time. Consequently, the number of participating hospitals was lower than originally planned (we expected 25 hospitals to participate) and those participating had little to no capacity to implement the intervention. The combination of barriers to implementation in general practices and hospitals resulted in the fact that no patient received in-hospital intervention components. Also, there were determinants to implementation that can be attributed to the programme itself. The programme is rated to be too elaborate and difficult to integrate into everyday processes. Concerning the assessment before admission, its benefit was rated lower by general practices than by hospitals. They did not have a direct positive benefit from it and might have seen their efforts wasted. Therefore, many general practices thus decided not to apply this intervention component and only include patients into the programme after hospital discharge. Intervention components such as the assessment for planning of follow-up care after discharge or the telephone monitoring were evaluated positively regarding its working mechanism, but the benefit is rated worth the effort by only approx. half of the participants. However, the effectiveness evaluation indicates that both the subgroup who received telephone monitoring (who are at high risk for readmission) and the subgroup without telephone monitoring (lower risk for readmission) profited from the intervention.

### Including primary care in admission and discharge management

Strong primary care is associated with utilisation of secondary care, such as hospital admission [37], readmission [38], admission due to ambulatory care sensitive conditions [39] or emergency services [40] and can support admission and discharge processes. Van Walraven et al. [41] and Leppert et al. [42] found that primary care follow-up after hospital discharge reduces risk of readmissions. Still, most interventions aiming at the reduction of readmissions take place before or after discharge [6]. Our intervention, however, covered the whole cross-sectoral care process beginning with pre-admission intervention components in general practice, followed by intervention components during the hospital stay and at hospital discharge and concluding in general practice. Including primary care into multi-component care transition-interventions thus represents an opportunity to contribute to the reduction of readmissions and further strengthen primary care [43,44,45,46]. This especially but not only applies to countries with traditionally weak primary care systems, such as Germany and other countries with social health insurance systems [47,48].

## Lessons learned

We have learned many lessons developing the VESPEERA programme, designing and conducting its evaluation and exchanging ideas and experiences within the project team and with all the participants from general practices and hospitals.

The simultaneity of implementing a new care programme and conducting a study makes acceptance more difficult, as time-consuming additional data collection and other efforts are necessary for the evaluation. Another consequence was that a randomised evaluation design deemed unfeasible. Complex interventions with the possibility of including patients in the study at different points in time face particular challenges due to the high workload of physicians in the reality of care. For future studies, we recommend to reduce the burden of data-collection on study participants when designing studies. Furthermore, we recommend planning with fewer study arms from the beginning. In addition, we recommend keeping the number of intervention components lower. Potential shares of the individual intervention components in the overall effect could then be determined based on subgroup analyses. Further comparison to non-GP-based care is recommended.

Addressing practice and policy, the following are our take-home messages:

- The VESPEERA programme hardly reached clinical decision makers in hospital. The parallelism of the implementation of a project and the mandatory implementation of legal regulations aiming at similar outcomes has inhibited the implementation of VESPEERA in hospitals. Incompatibility of our programme with the information systems in hospitals further added to these barriers. Our collaboration with support staff in hospitals did not yield much impact.
- The focus on patients of one large health insurer (covering 45% of the population in that region), who were referred by GPs, may have been too narrow to be of interest to hospitals. In Germany, many patients enter hospital by admission of specialist physicians or as emergency cases, not as planned hospital admissions by general practitioners.
- ‘Talking to each other’ is the be-all and end-all in cross-sectoral care. The workshops with the stakeholders to develop the intervention components and the feedback meetings have, in our opinion, moved a lot and increased acceptance for the problems and challenges of the respective ‘other side’ - quite independently of the VESPEERA programme. Regional initiatives such as joint quality circles and regulars’ tables, as already established among ambulatory physicians, could help to stabilise communication.
- When co-designing care programmes with all relevant stakeholders, care should be taken that, even though efforts are made to consider the requirements and needs of all, the intervention does not become too comprehensive. Another possibility would be to make parts of the programme mandatory and others optional.
- Admission and discharge management should not be reduced to the times of admission and discharge. The provision of more information by GPs at admission, for example by means of a mandatory and structured admission letter, should be promoted. In addition, GPs should be more involved in follow-up care. Admission and discharge management should become a shared cross-sectoral task and early planning of hospital discharge should start at the time of admission.
- Risk assessments at discharge provided by either the discharging hospital or the GP providing follow-up care, for example by means of the HOSPITAL score, can help identify patients at risk of unplanned readmission. These patients can then be closely monitored and taken care of, for example through telephone follow-up.
- For future studies, we recommend planning with fewer study arms from the beginning. If sample sizes are sufficiently large, the effect of intervention components could then be separated by conducting sub group analyses.

## Conclusion

The results of our study on development, implementation and evaluation of an admission, discharge and follow-up intervention emphasise the relevance of treating admission and discharge management as a cross-sectoral task. Patients can possibly benefit from the intervention. It is of high importance to not only leave this responsibility to the inpatient sector but to involve primary care teams in both pre and post hospital care.

## Data Accessibility Statements

Datasets are available upon reasonable request with the data owner.

## Additional File

10.5334/ijic.6534.s1 TIDieR list, Additional Files 1–10. Tables on the results of the effectiveness analysis and results of the quantitative survey.

## Figures and Tables

**Table:** 

CCI	Charlson Comorbidity Index
GP	General Practitioner
OR	Odds Ratio
SD	Standard Deviation
VERAH	Care Assistant in General Practice (*Versorgungsassistentin in der Hausarztpraxis*)

## References

[1] Forster AJ, Clark HD, Menard A, Dupuis N, Chernish R, Chandok N, et al. Adverse events among medical patients after discharge from hospital. Canadian Medical Association Journal. 2004; 170(3): 345–9. 14757670 PMC331384

[2] Moore C, Wisnivesky J, Williams S, McGinn T. Medical errors related to discontinuity of care from an inpatient to an outpatient setting. Journal of General Internal Medicine. 2003; 18(8): 646–51. DOI: 10.1046/j.1525-1497.2003.20722.x 12911647 PMC1494907

[3] Kripalani S, LeFevre F, Phillips CO, Williams MV, Basaviah P, Baker DW. Deficits in communication and information transfer between hospital-based and primary care physicians: implications for patient safety and continuity of care. JAMA. 2007; 297(8): 831–41. DOI: 10.1001/jama.297.8.831 17327525

[4] van Walraven C, Seth R, Austin PC, Laupacis A. Effect of discharge summary availability during post-discharge visits on hospital readmission. Journal of General Internal Medicine. 2002; 17(3): 186–92. DOI: 10.1046/j.1525-1497.2002.10741.x 11929504 PMC1495026

[5] Hesselink G, Flink M, Olsson M, Barach P, Dudzik-Urbaniak E, Orrego C, et al. Are patients discharged with care? A qualitative study of perceptions and experiences of patients, family members and care providers. BMJ Quality & Safety. 2012; 21(Suppl 1): i39–49. DOI: 10.1136/bmjqs-2012-001165 23118410

[6] Straßner C, Hoffmann M, Forstner J, Roth C, Szecsenyi J, Wensing M. Interventions to Improve Hospital Admission and Discharge Management: An Umbrella Review of Systematic Reviews. Quality management in health care. 2020; 29(2): 67–75. DOI: 10.1097/QMH.0000000000000244 32224790

[7] Hesselink G, Zegers M, Vernooij-Dassen M, Barach P, Kalkman C, Flink M, et al. Improving patient discharge and reducing hospital readmissions by using Intervention Mapping. BMC health services research. 2014; 14: 389. DOI: 10.1186/1472-6963-14-389 25218406 PMC4175223

[8] Leppin AL, Gionfriddo MR, Kessler M, Brito JP, Mair FS, Gallacher K, et al. Preventing 30-day hospital readmissions: a systematic review and meta-analysis of randomized trials. JAMA internal medicine. 2014; 174(7): 1095–107. DOI: 10.1001/jamainternmed.2014.1608 24820131 PMC4249925

[9] Hansen LO, Young RS, Hinami K, Leung A, Williams MV. Interventions to reduce 30-day rehospitalization: a systematic review. Annals of internal medicine. 2011; 155(8): 520–8. DOI: 10.7326/0003-4819-155-8-201110180-00008 22007045

[10] Mistiaen P, Francke AL, Poot E. Interventions aimed at reducing problems in adult patients discharged from hospital to home: a systematic meta-review. BMC Health Services Research. 2007; 7: 47. DOI: 10.1186/1472-6963-7-47 17408472 PMC1853085

[11] Scott IA. Preventing the rebound: improving care transition in hospital discharge processes. Australian health review: a publication of the Australian Hospital Association. 2010; 34(4): 445–51. DOI: 10.1071/AH09777 21108906

[12] Schlette S, Lisac M, Blum K. Integrated primary care in Germany: the road ahead. International Journal of Integrated Care. 2009; 9: e14. DOI: 10.5334/ijic.311 19513180 PMC2691944

[13] Kringos DS, Boerma WGW, van der Zee J, Groenewegen PP. Political, cultural and economic foundations of primary care in Europe. Social science & medicine (1982). 2013; 99: 9–17. DOI: 10.1016/j.socscimed.2013.09.017 24355465

[14] Freytag A, Biermann J, Ochs A, Lux G, Lehmann T, Ziegler J, et al. The Impact of GP-Centered Healthcare. Deutsches Arzteblatt international. 2016; 113(47): 791–8. DOI: 10.3238/arztebl.2016.0791 28043322 PMC5240023

[15] Wensing M, Kolle PK, Szecsenyi J, Stock C, Laux G. Effects of a program to strengthen general practice care on hospitalisation rates: a comparative observational study. Scandinavian Journal of Primary Health Care. 2018; 36(2): 109–14. DOI: 10.1080/02813432.2018.1459429 29623749 PMC6066294

[16] Wensing M, Szecsenyi J, Stock C, Kaufmann Kolle P, Laux G. Evaluation of a program to strengthen general practice care for patients with chronic disease in Germany. BMC Health Services Research. 2017; 17(1): 62. DOI: 10.1186/s12913-017-2000-2 28109281 PMC5251235

[17] Brooke BS, Stone DH, Cronenwett JL, Nolan B, DeMartino RR, MacKenzie TA, et al. Early primary care provider follow-up and readmission after high-risk surgery. JAMA surgery. 2014; 149(8): 821–8. DOI: 10.1001/jamasurg.2014.157 25074237 PMC4287962

[18] Balaban RB, Weissman JS, Samuel PA, Woolhandler S. Redefining and redesigning hospital discharge to enhance patient care: a randomized controlled study. Journal of General Internal Medicine. 2008; 23(8): 1228–33. DOI: 10.1007/s11606-008-0618-9 18452048 PMC2517968

[19] Lee KH, Low LL, Allen J, Barbier S, Ng LB, Ng MJM, et al. Transitional care for the highest risk patients: findings of a randomised control study. International Journal of Integrated Care. 2015; 15: e039. DOI: 10.5334/ijic.2003 27118956 PMC4843175

[20] Goodwin N. Understanding Integrated Care. International Journal of Integrated Care. 2016; 16(4): 6. DOI: 10.5334/ijic.2530 PMC5354214 28316546

[21] AQUA – Institut für angewandte Qualitätsförderung und Forschung im Gesundheitswesen GmbH. Entlassungsmanagement – Konzeptskizze für ein Qualitätssicherungsverfahren. Göttingen; 2015. [cited 2022 Feb 25]. Available from: https://sqg.de/sqg/upload/CONTENT/Neue-Verfahren/Entlassungsmanagement/Bericht_Konzeptskizze_Entlassungsmanagement.pdf.

[22] Erweitertes Bundesschiedsamt für die vertragsärztliche Versorgung. Rahmenvertrag über ein Entlassmanagement beim Übergang in die Versorgung nach Krankenhausbehandlung nach § 39 Abs. 1a S. 9 SGB V (Rahmenvertrag Entlassmanagement); 2016.

[23] Blümel M, Spranger A, Achstetter K, Maresso A, Busse R. Germany: Health System Review. Health systems in transition. 2020; 22(6): 1–272. 34232120

[24] Möller K-H, Makoski K. Der Arztbrief – Rechtliche Rahmenbedingungen. KrV Kranken- und Pflegeversicherung 2015; 5. DOI: 10.37307/j.2193-5661.2015.05.05

[25] Forstner J, Bossert J, Weis A, Litke N, Strassner C, Szecsenyi J, Wensing M. The role of personalised professional relations across care sectors in achieving high continuity of care. BMC Family Practice. 2021; 22(1): 72. DOI: 10.1186/s12875-021-01418-8 33849453 PMC8045382

[26] Freund T, Everett C, Griffiths P, Hudon C, Naccarella L, Laurant M. Skill mix, roles and remuneration in the primary care workforce: Who are the healthcare professionals in the primary care teams across the world? International journal of nursing studies. 2015; 52(3): 727–43. DOI: 10.1016/j.ijnurstu.2014.11.014 25577306

[27] Mergenthal K, Beyer M, Gerlach FM, Güthlin C. Wie werden Delegationskonzepte in Hausarztpraxen ausgestaltet? Eine Analyse am Beispiel der VERAH in der HzV. Zeitschrift für Allgemeinmedizin. 2016; 92(10): 402–7. DOI: 10.3238/zfa.2016.0402-0407

[28] Donzé JD, Williams MV, Robinson EJ, Zimlichman E, Aujesky D, Vasilevskis EE, et al. International Validity of the HOSPITAL Score to Predict 30-Day Potentially Avoidable Hospital Readmissions. JAMA internal medicine. 2016; 176(4): 496–502. DOI: 10.1001/jamainternmed.2015.8462 26954698 PMC5070968

[29] Forstner J, Litke N, Weis A, Straßner C, Szecsenyi J, Wensing M. How to fall into a new routine: factors influencing the implementation of an admission and discharge programme in hospitals and general practices. BMC Health Services Research. 2022; 22(1): 1289. DOI: 10.1186/s12913-022-08644-5 36284324 PMC9598008

[30] Forstner J, Kunz A, Straßner C, Uhlmann L, Kuemmel S, Szecsenyi J, Wensing M. Improving Continuity of Patient Care Across Sectors: Study Protocol of the Process Evaluation of a Quasi-Experimental Multi-Centre Study Regarding an Admission and Discharge Model in Germany (VESPEERA). BMJ Open. 2019; 9(11): e031245. DOI: 10.1136/bmjopen-2019-031245 PMC6858220 31722944

[31] Dimick JB, Ryan AM. Methods for evaluating changes in health care policy: the difference-in-differences approach. JAMA. 2014; 312(22): 2401–2. DOI: 10.1001/jama.2014.16153 25490331

[32] Kontopantelis E, Doran T, Springate DA, Buchan I, Reeves D. Regression based quasi-experimental approach when randomisation is not an option: interrupted time series analysis. BMJ (Clinical research ed.) 2015; 350: h2750. DOI: 10.1136/bmj.h2750 26058820 PMC4460815

[33] Hoffmann TC, Glasziou PP, Boutron I, Milne R, Perera R, Moher D, et al. Better reporting of interventions: template for intervention description and replication (TIDieR) checklist and guide. BMJ. 2014; 348: g1687. DOI: 10.1136/bmj.g1687 24609605

[34] Forstner J, Straßner C, Kunz A, Uhlmann L, Freund T, Peters-Klimm F, et al. Improving continuity of patient care across sectors: study protocol of a quasi-experimental multi-centre study regarding an admission and discharge model in Germany (VESPEERA). BMC health services research. 2019; 19(1): 206. DOI: 10.1186/s12913-019-4022-4 30925879 PMC6441227

[35] Huang Y-q, Gou R, Diao Y-s, Yin Q-h, Fan W-x, Liang Y-p, et al. Charlson comorbidity index helps predict the risk of mortality for patients with type 2 diabetic nephropathy. Journal of Zhejiang University SCIENCE B. 2014; 15(1): 58–66. DOI: 10.1631/jzus.B1300109 24390745 PMC3891119

[36] Facchinetti G, D’Angelo D, Piredda M, Petitti T, Matarese M, Oliveti A, Marinis MG de. Continuity of care interventions for preventing hospital readmission of older people with chronic diseases: A meta-analysis. International journal of nursing studies. 2020; 101: 103396. DOI: 10.1016/j.ijnurstu.2019.103396 31698168

[37] Sawicki OA, Mueller A, Klaaßen-Mielke R, Glushan A, Gerlach FM, Beyer M, et al. Strong and sustainable primary healthcare is associated with a lower risk of hospitalization in high risk patients. Scientific Reports. 2021; 11(1): 4349. DOI: 10.1038/s41598-021-83962-y 33623130 PMC7902818

[38] Wensing M, Szecsenyi J, Laux G. Continuity in general practice and hospitalization patterns: an observational study. BMC Family Practice. 2021; 22(1): 21. DOI: 10.1186/s12875-020-01361-0 33446104 PMC7809859

[39] van Loenen T, van den Berg MJ, Westert GP, Faber MJ. Organizational aspects of primary care related to avoidable hospitalization: a systematic review. Family practice. 2014; 31(5): 502–16. DOI: 10.1093/fampra/cmu053 25216664

[40] Huntley A, Lasserson D, Wye L, Morris R, Checkland K, England H, et al. Which features of primary care affect unscheduled secondary care use? A systematic review. BMJ Open. 2014; 4(5): e004746. DOI: 10.1136/bmjopen-2013-004746 PMC4039790 24860000

[41] van Walraven C, Taljaard M, Etchells E, Bell CM, Stiell IG, Zarnke K, Forster AJ. The independent association of provider and information continuity on outcomes after hospital discharge: Implications for hospitalists. Journal of hospital medicine. 2010; 5(7): 398–405. DOI: 10.1002/jhm.716 20845438

[42] Leppert MH, Sillau S, Lindrooth RC, Poisson SN, Campbell JD, Simpson JR. Relationship between early follow-up and readmission within 30 and 90 days after ischemic stroke. Neurology. 2020; 94(12): e1249–e1258. DOI: 10.1212/WNL.0000000000009135 32079738 PMC7274933

[43] Bricard D, Or Z. Impact of early primary care follow-up after discharge on hospital readmissions. The European journal of health economics: HEPAC: health economics in prevention and care. 2019; 20(4): 611–23. DOI: 10.1007/s10198-018-1022-y 30600468

[44] Saluja S, Hochman M, Bourgoin A, Maxwell J. Primary Care: the New Frontier for Reducing Readmissions. Journal of General Internal Medicine. 2019; 34(12): 2894–7. DOI: 10.1007/s11606-019-05428-2 31621049 PMC6854170

[45] Spencer RA, Singh Punia H. A scoping review of communication tools applicable to patients and their primary care providers after discharge from hospital. Patient education and counseling. 2021; 104(7): 1681–703. DOI: 10.1016/j.pec.2020.12.010 33446366

[46] Spivack SB, DeWalt D, Oberlander J, Trogdon J, Shah N, Meara E, et al. The Association of Readmission Reduction Activities with Primary Care Practice Readmission Rates. Journal of General Internal Medicine. 2022; 37(12): 3005–12. DOI: 10.1007/s11606-021-07005-y 34258724 PMC9485329

[47] Groenewegen PP, Dourgnon P, Greß S, Jurgutis A, Willems S. Strengthening weak primary care systems: steps towards stronger primary care in selected Western and Eastern European countries. Health policy (Amsterdam, Netherlands). 2013; 113(1–2): 170–9. DOI: 10.1016/j.healthpol.2013.05.024 23895880

[48] Kringos DS, Boerma W, van der Zee J, Groenewegen P. Europe’s strong primary care systems are linked to better population health but also to higher health spending. Health affairs (Project Hope). 2013; 32(4): 686–94. DOI: 10.1377/hlthaff.2012.1242 23569048

