# Supplementary material for: Hospital Admission and Discharge: Lessons Learned from a Large Programme in Southwest Germany
Source: Int J Integr Care. 2023 Jan 27;23(1):4. doi: 10.5334/ijic.6534 (PMC9881439; doi:10.5334/ijic.6534)
Supplement: TIDieR list, Additional Files 1–10. — Tables on the results of the effectiveness analysis and results of the quantitative survey. [file ijic-23-1-6534-s1.zip › s1-ijic-6534_forstner/6534-24595-1-SP.docx]

Additional File 1

Characteristics of the study population

| Variables | intervention | control | Total |
| --- | --- | --- | --- |
|  | (N = 371) | (N = 371) | (N = 742) |
| Age |  |  |  |
| mean | 69 | 70 | 70 |
| sd* | 16 | 16 | 16 |
| median | 74 | 72 | 74 |
| Q1 - Q3 | 59 -- 81 | 60 -- 82 | 59 -- 82 |
| min - max | 18 -- 96 | 18 -- 99 | 18 -- 99 |
| Gender |  |  |  |
| male | 182 (49%) | 183 (49%) | 365 (49%) |
| female | 189 (51%) | 188 (51%) | 377 (51%) |
| admissions |  |  |  |
| mean | 2.7 | 2.7 | 2.7 |
| sd* | 2.3 | 2.2 | 2.2 |
| median | 2 | 2 | 2 |
| Q1 - Q3 | 1 -- 4 | 1 -- 3 | 1 -- 3 |
| min - max | 1 -- 17 | 1 -- 15 | 1 -- 17 |
| CCI** at first admission |  |  |  |
| mean | 3.6 | 3.4 | 3.5 |
| sd* | 2.8 | 2.8 | 2.8 |
| median | 3 | 3 | 3 |
| Q1 - Q3 | 1 -- 6 | 1 -- 6 | 1 -- 6 |
| min - max | 0 -- 13 | 0 -- 12 | 0 -- 13 |
| Most common diagnoses of the overall study population (n) | |  |  |
|  | heart failure (54) | heart failure (50) | heart failure (104) |
|  | COPD*** (40) | Hypertension (25) | COPD*** (47) |
|  | lung cancer (27) | acute myocardial infarction (24) | acute myocardial infarction (45) |
|  | atrial fibrillation (24) | angina pectoris (22) | angina pectoris (44) |
|  | angina pectoris (22) | cerebral infarction (21) | atrial fibrillation (43) |
| Most common diagnoses of patients with readmission (n) | |  |  |
|  | heart failure (41) | heart failure (36) | heart failure (77) |
|  | COPD*** (34) | Hypertension (19) | COPD*** (38) |
|  | lung cancer (C34) | chronic ischemic heart disease (15) | chronic ischemic heart disease (31) |
|  | chronic ischemic heart disease (16) | angina pectoris (14) | lung cancer (30) |
|  | atrial fibrillation (16) | atherosclerosis (13) | angina pectoris (27) |
| *sd: standard deviation  **CCI: Charlson Comorbidity Index  ***COPD: chronic obstructive pulmonary disease | |  |  |
