# Supplementary material for: Hospital Admission and Discharge: Lessons Learned from a Large Programme in Southwest Germany
Source: Int J Integr Care. 2023 Jan 27;23(1):4. doi: 10.5334/ijic.6534 (PMC9881439; doi:10.5334/ijic.6534)
Supplement: TIDieR list, Additional Files 1–10. — Tables on the results of the effectiveness analysis and results of the quantitative survey. [file ijic-23-1-6534-s1.zip › s1-ijic-6534_forstner/6534-24596-1-SP.docx]

Additional File 2

Descriptive results of the outcomes

| Group | Rate Pre-intervention [95% CI*] | Rate Post-intervention [95% CI*] |
| --- | --- | --- |
| Readmission within 90 days | | |
| intervention | 15.8% [10.6%; 22.9%] | 13.3% [11.1%; 15.9%] |
| controls | 9.5% [5.1%; 17%] | 13.0% [10.8%; 15.6%] |
| Readmission within 30 days | | |
| intervention | 4.5% [2.1%; 9.5%] | 6.9% [5.3%; 8.9%] |
| controls | 5.3% [2.3%; 11.7%] | 5.2% [3.8%; 7%] |
| Admission due to ACSC** | | |
| intervention | 4.5% [2.1%; 9.5%] | 3.9% [2.7%; 5.5%] |
| controls | 3.2% [1.1%; 8.9%] | 4.0% [2.9%; 5.7%] |
| Delayed prescription of medication | | |
| intervention | 28.1% [20.9%; 36.7%] | 19.6% [16.8%; 22.7%] |
| controls | 21.2% [13.8%; 31%] | 24.0% [21%; 27.3%] |
| Delayed prescription of medical aids and appliances | | |
| intervention | 69.2% [55.7%; 80.1%] | 56.7% [51%; 62.3%] |
| controls | 73.5% [56.9%; 85.4]% | 65.8% [60.2%; 70.9%] |
| Delayed referral to rehabilitation therapeutics | | |
| intervention | 74.3% [57.9%; 85.8%] | 74.2% [67.5%; 79.9%] |
| controls | 77.3% [56.6%; 89.9%] | 68.8% [61.5%; 75.2%] |
| Emergency/ rescue services | | |
| intervention | 32.3% [25%; 40.7%] | 30.3% [27.1%; 33.6%] |
| controls | 29.5% [21.2%; 39.3%] | 33.9% [30.7%; 37.4%] |
| *CI: confidence interval  **ACSC: ambulatory care sensitive conditions | | |
