# Supplementary material for: Hospital Admission and Discharge: Lessons Learned from a Large Programme in Southwest Germany
Source: Int J Integr Care. 2023 Jan 27;23(1):4. doi: 10.5334/ijic.6534 (PMC9881439; doi:10.5334/ijic.6534)
Supplement: TIDieR list, Additional Files 1–10. — Tables on the results of the effectiveness analysis and results of the quantitative survey. [file ijic-23-1-6534-s1.zip › s1-ijic-6534_forstner/6534-24597-1-SP.docx]

Additional File 3

Results of the statistical analysis

| Comparison | Odds Ratio | Confidence interval | p-value |
| --- | --- | --- | --- |
| Readmission within 90 days | 0.662 | [0.261, 1.680] | 0.38518 |
| Readmission within 30 days | 2.129 | [0.547, 8.285] |  |
| Admission due to ACSC* | 0.467 | [0.156, 1.399] |  |
| Delayed prescription of medication | 0.530 | [0.263, 1.068] |  |
| Delayed prescription of medical aids and appliances | 0.843 | [0.304, 2.337] |  |
| Delayed referral to rehabilitation therapeutics | 1.535 | [0.404, 5.830] |  |
| Emergency or rescue services | 0.696 | [0.328, 1.478] |  |
| *ACSC: ambulatory care sensitive conditions | |  |  |
