# Supplementary material for: Hospital Admission and Discharge: Lessons Learned from a Large Programme in Southwest Germany
Source: Int J Integr Care. 2023 Jan 27;23(1):4. doi: 10.5334/ijic.6534 (PMC9881439; doi:10.5334/ijic.6534)
Supplement: TIDieR list, Additional Files 1–10. — Tables on the results of the effectiveness analysis and results of the quantitative survey. [file ijic-23-1-6534-s1.zip › s1-ijic-6534_forstner/6534-24598-1-SP.docx]

Additional File 4

Results of the subgroup analysis

| Comparison | Odds Ratio | Confidence interval |
| --- | --- | --- |
| No telephone monitoring | 0.553 | [0.218, 1.406] |
| Telephone monitoring | 0.695 | [0.204, 2.367] |
| Age < 65 years | 0.661 | [0.125, 3.488] |
| Age at least 65 years | 0.464 | [0.129, 1.662] |
| Low CCI* | 3.808 | [0.73, 19.859] |
| Medium CCI* | 0.712 | [0.093, 5.475] |
| High CCI* | 0.113 | [0.013, 0.997] |
| *CCI: Charlson Comorbidity Index | |  |
