# Supplementary material for: Hospital Admission and Discharge: Lessons Learned from a Large Programme in Southwest Germany
Source: Int J Integr Care. 2023 Jan 27;23(1):4. doi: 10.5334/ijic.6534 (PMC9881439; doi:10.5334/ijic.6534)
Supplement: TIDieR list, Additional Files 1–10. — Tables on the results of the effectiveness analysis and results of the quantitative survey. [file ijic-23-1-6534-s1.zip › s1-ijic-6534_forstner/6534-24599-1-SP.docx]

Additional File 5

Characteristics of the study population of the quantitative survey

|  | mean (SD) | n (%) | n |
| --- | --- | --- | --- |
| gender *(male)* |  | 16 (24,2) | 66 |
| Age | 48 (11.4) |  | 63 |
| Work experience *(in years)* | 22.8 (11) |  | 62 |
| County of the organisation *(urban)* |  | 29 (43,9) | 66 |
| organisation *(general practice)* |  | 50 (73,5) | 68 |
| Size of the general practice *(patients per quarter year)* | 1660.4 (640.1) |  | 48 |
| Size of the hospital *(beds per department)* | 378.3 (536.4) |  | 17 |
| Number of [BLINDED] patients | 9.1 (21) |  | 62 |
