# Supplementary material for: Hospital Admission and Discharge: Lessons Learned from a Large Programme in Southwest Germany
Source: Int J Integr Care. 2023 Jan 27;23(1):4. doi: 10.5334/ijic.6534 (PMC9881439; doi:10.5334/ijic.6534)
Supplement: TIDieR list, Additional Files 1–10. — Tables on the results of the effectiveness analysis and results of the quantitative survey. [file ijic-23-1-6534-s1.zip › s1-ijic-6534_forstner/6534-24600-1-SP.docx]

Additional File 6

Self-reported intervention fidelity

| [BLINDED] component | n(%) | n |
| --- | --- | --- |
| Assessment before admission was used at least once | 33 (49.3) | 67 |
| Admission letter was used at least once | 32 (47.8) | 67 |
| Patient brochure was handed out at least once *(general practice only)* | 31 (68.9) | 45 |
| Familiar with the content of the patient brochure | 34 (77.3) | 44 |
| HOSPITAL score was collected at least once | 29 (44.6) | 65 |
| Telephonic discharge conversation was used at least once | 11 (16.9) | 65 |
| At least one case is known in which the patient discharge information was used *(hospital only)* | 2 (8.7) | 23 |
| Assessment for planning of follow-up care after discharge was used at least once *(general practice only)* | 38 (77.6) | 49 |
| Telephone monitoring was used at least once *(general practice only)* | 34 (70.8) | 48 |
| Fidelity (Score between 0 and 1); mean(SD) | 0.4 (0.4) | 68 |
