# Supplementary material for: Hospital Admission and Discharge: Lessons Learned from a Large Programme in Southwest Germany
Source: Int J Integr Care. 2023 Jan 27;23(1):4. doi: 10.5334/ijic.6534 (PMC9881439; doi:10.5334/ijic.6534)
Supplement: TIDieR list, Additional Files 1–10. — Tables on the results of the effectiveness analysis and results of the quantitative survey. [file ijic-23-1-6534-s1.zip › s1-ijic-6534_forstner/6534-24601-1-SP.docx]

Additional File7

Working mechanism of the [BLINDED] components

|  | Not at all true | Rather not true | Partly true | Rather true | Very true | mean (SD) | n |
| --- | --- | --- | --- | --- | --- | --- | --- |
| **Assessment before admission/ admission letter** |  |  |  |  |  |  |  |
| … helps to get new relevant information about the patients. | 3 (5.3 %) | 3 (5.3 %) | 13 (22.8 %) | 27 (47.4 %) | 11 (19.3 %) | 3.7 (1) | 57 |
| … helps to complete the documentation of patient data in my practice/ clinic. | 3 (5.3 %) | 5 (8.8 %) | 16 (28.15 %) | 25 (43.9 %) | 8 (14 %) | 3.5 (1) | 57 |
| … leads to a clearer communication of the indication for admission. | 3 (5.2 %) | 4 (6.9 %) | 13 (22.4 %) | 27 (46.6 %) | 11 (19 %) | 3.7 (1) | 58 |
| … helps to get a better understanding of the patient's social situation before admission. | 2 (3.4 %) | 3 (5.2 %) | 7 (12.1 %) | 31 (53.4 %) | 15 (25.9 %) | 3.9 (1) | 58 |
| … leads to an acceleration of the admission process in the clinic. | 6 (10.2 %) | 22 (37.3 %) | 20 (33.9 %) | 8 (13.6 %) | 3 (5.1 %) | 2.7 (1) | 59 |
| … contains information relevant to the patient's discharge planning. | 0 | 6 (10.5 %) | 23 (40.4 %) | 22 (38.6 %) | 6 (10.5 %) | 3.5 (0.8) | 57 |
| … contains important information for the nursing staff. | 2 (3.4 %) | 1 (1.7 %) | 18 (31 %) | 32 (55.2 %) | 5 (8.6 %) | 3.6 (0.8) | 58 |
| … contains important information for clinicians | 2 (3.4 %) | 0 | 16 (27.6 %) | 32 (55.2 %) | 8 (13.8 %) | 3.8 (0.8) | 58 |
| … contains important information for the social service/case management. | 2 (3.4 %) | 1 (1.7 %) | 6 (10.3 %) | 40 (69 %) | 9 (15.5 %) | 3.9 (0.8) | 58 |
| Overall, the benefit of the admission letter exceeds the effort. | 4 (6.8 %) | 8 (13.6 %) | 18 (30.5 %) | 23 (39 %) | 6 (10.2 %) | 3.3 (1.1) | 59 |

|  | Not at all true | Rather not true | Partly true | Rather true | Very true | mean (SD) | n |
| --- | --- | --- | --- | --- | --- | --- | --- |
| **Patient brochure** |  |  |  |  |  |  |  |
| … helps motivate patients to claim their rights during the hospital stay. | 2 (6.1 %) | 4 (12.1 %) | 7 (21.2 %) | 18 (54.5 %) | 2 (6.1 %) | 3.4 (1) | 33 |
| … helps motivate patients to actively contribute - within their means - to their recovery process. | 2 (6.1 %) | 4 (12.1 %) | 4 (12.1 %) | 20 (60.6 %) | 3 (9.1 %) | 3.6 (1) | 33 |
| Overall, the patient brochure helps to prepare patients for a hospital stay. | 3 (8.8 %) | 2 (5.9 %) | 6 (17.6 %) | 15 (44.1 %) | 8 (23.5 %) | 3.7 (1.2) | 34 |
|  | Not at all true | Rather not true | Partly true | Rather true | Very true | mean (SD) | n |
| **HOSPITAL-Score** |  |  |  |  |  |  |  |
| … is easy to collect. | 2 (3.8 %) | 5 (9.6 %) | 21 (40.4 %) | 22 (42.3 %) | 2 (3.8 %) | 3.3 (0.9) | 52 |
| … helps identify patients at increased risk of readmission. | 2 (3.8 %) | 7 (13.2 %) | 18 (34 %) | 20 (37.7 %) | 6 (11.3 %) | 3.4 (1) | 53 |
| … helps to identify patients with increased support needs. | 1 (1.9 %) | 9 (17 %) | 15 (28.3 %) | 24 (45.3 %) | 4 (7.5 %) | 3.4 (0.9) | 53 |
| … helps to identify patients for inclusion in the telephone monitoring. | 1 (1.9 %) | 5 (9.6 %) | 19 (36.5 %) | 22 (42.3 %) | 5 (9.6 %) | 3.5 (0.9) | 52 |
| Overall, the benefit of the HOSPITAL-Score exceeds the effort. | 3 (5.6 %) | 7 (13 %) | 24 (44.4 %) | 16 (29.6 %) | 4 (7.4 %) | 3.2 (1) | 54 |

|  | Not at all true | Rather not true | Partly true | Rather true | Very true | mean (SD) | n |
| --- | --- | --- | --- | --- | --- | --- | --- |
| **Telephonic discharge conversation** |  |  |  |  |  |  |  |
| … promotes general cooperation between hospital and general practice. | 1 (1.7 %) | 5 (8.3 %) | 9 (15 %) | 31 (51.7 %) | 14 (23.3 %) | 3.9 (0.9) | 60 |
| … is helpful for complex or vulnerable patients. | 1 (1.7 %) | 2 (3.4 %) | 8 (13.6 %) | 32 (54.2 %) | 16 (27.1 %) | 4 (0.8) | 59 |
| … should generally be carried out for all patients/ is helpful for all patients. | 5 (8.5 %) | 10 (16.9 %) | 20 (33.9 %) | 18 (30.5 %) | 6 (10.2 %) | 3.2 (1.1) | 59 |
| Overall, the benefit of the telephone discharge conversation exceeds the effort. | 3 (5.4 %) | 5 (8.9 %) | 22 (39.3 %) | 19 (33.9 %) | 7 (12.5 %) | 3.4 (1) | 56 |

|  | Not at all true | Rather not true | Partly true | Rather true | Very true | mean (SD) | n |
| --- | --- | --- | --- | --- | --- | --- | --- |
| **Patient discharge information** |  |  |  |  |  |  |  |
| … helps to inform patients about what they themselves can contribute to effective follow-up treatment. | 0 | 1 (5.9 %) | 5 (29.4 %) | 9 (52.9 %) | 2 (11.8 %) | 3.7 (0.8) | 17 |
| … helps prepare patients for the first days after discharge from hospital. | 0 | 1 (5.9 %) | 2 (11.8 %) | 13 (76.5 %) | 1 (5.9 %) | 3.8 (0.6) | 17 |
| … is a useful addition to the discharge conversation. | 0 | 1 (5.9 %) | 4 (23.5 %) | 8 (47.1 %) | 4 (23.5 %) | 3.9 (0.9) | 17 |
| … provides the patient with the right contact persons. | 0 | 1 (5.9 %) | 5 (29.4 %) | 7 (41.2 %) | 4 (23.5 %) | 3.8 (0.9) | 17 |
| Overall, the benefit of the patient discharge information of the [BLINDED] project exceeds the effort. | 0 | 1 (5.9 %) | 6 (35.3 %) | 8 (47.1 %) | 2 (11.8 %) | 3.7 (0.8) | 17 |

|  | Not at all true | Rather not true | Partly true | Rather true | Very true | mean (SD) | n |
| --- | --- | --- | --- | --- | --- | --- | --- |
| **Assessment for planning of follow-up care after discharge** |  |  |  |  |  |  |  |
| … helps to give the patient space to tell about their hospital stay. | 1 (2.9 %) | 1 (2.9 %) | 5 (14.3 %) | 15 (42.9 %) | 13 (37.1 %) | 4.1 (1) | 35 |
| … helps me to get an overview of the patient's medication after discharge. | 1 (2.9 %) | 1 (2.9 %) | 4 (11.4 %) | 18 (51.4 %) | 11 (31.4 %) | 4.1 (0.9) | 35 |
| … helps to think of all the measures that need to be initiated after discharge. | 0 | 1 (2.9 %) | 3 (8.6 %) | 21 (60 %) | 10 (28.6 %) | 4.1 (0.7) | 35 |
| … helps to identify further care and treatment needs at an early stage. | 0 | 2 (5.7 %) | 5 (14.3 %) | 20 (57.1 %) | 8 (22.9 %) | 4 (0.8) | 35 |
| Overall, the benefit of the assessment for planning of follow-up care after discharge exceeds the effort. | 2 (5.6 %) | 4 (11.1 %) | 11 (30.6 %) | 14 (38.9 %) | 5 (13.9 %) | 3.5 (1.1) | 36 |

|  | Not at all true | Rather not true | Partly true | Rather true | Very true | mean (SD) | n |
| --- | --- | --- | --- | --- | --- | --- | --- |
| **Telephone monitoring** |  |  |  |  |  |  |  |
| … helps to check the patient's adherence to therapy. | 1 (2.8 %) | 3 (8.3 %) | 1 (2.8 %) | 27 (75 %) | 4 (11.1 %) | 3.8 (0.9) | 36 |
| … helps to identify further care and treatment needs. | 1 (2.8 %) | 1 (2.8 %) | 5 (13.9 %) | 24 (66.7 %) | 5 (13.9 %) | 3.9 (0.8) | 36 |
| … helps to avoid early readmissions to hospital. | 1 (2.8) | 3 (8.3 %) | 12 (33.3 %) | 18 (50 %) | 2 (5.6 %) | 3.5 (0.8) | 36 |
| … helps with complex or vulnerable patients. | 1 (2.9 %) | 1 (2.9 %) | 10 (28.6 %) | 19 (54.3 %) | 4 (11.4 %) | 3.7 (0.8) | 35 |
| … would also be helpful for patients without a high HOSPITAL score. | 1 (2.8 %) | 4 (11.1 %) | 11 (130.6 %) | 15 (41.7 %) | 5 (13.9 %) | 3.5 (1) | 36 |
| Overall, the benefit of the telephone monitoring exceeds the effort. | 1 (2.7 %) | 4 (10.8 %) | 11 (29.7 %) | 17 (45.9 %) | 4 (10.8 %) | 3.5 (0.9) | 37 |
