# Supplementary material for: Hospital Admission and Discharge: Lessons Learned from a Large Programme in Southwest Germany
Source: Int J Integr Care. 2023 Jan 27;23(1):4. doi: 10.5334/ijic.6534 (PMC9881439; doi:10.5334/ijic.6534)
Supplement: TIDieR list, Additional Files 1–10. — Tables on the results of the effectiveness analysis and results of the quantitative survey. [file ijic-23-1-6534-s1.zip › s1-ijic-6534_forstner/6534-24602-1-SP.docx]

Additional File 8

Contextual determinants to the implementation of the [BLINDED] programme

|  | Not at all true | Rather not true | Partly true | Rather true | Very true | mean (SD) | n |
| --- | --- | --- | --- | --- | --- | --- | --- |
| **Networks** |  |  |  |  |  |  |  |
| The majority of patients in the region go to the same hospital. | 0 | 12 (18.5 %) | 15 (23.1 %) | 29 (44.6 %) | 9 (13.8 %) | 3.5 (1) | 65 |
| The hospital where most of the patients go is the only hospital in the region. | 25 (38.5 %) | 27 (41.5 %) | 8 (12.3 %) | 5 (7.7 %) | 0 | 1.9 (0.9) | 65 |
| I have been working with the local hospital/GPs in the region for many years. | 3 (4.7 %) | 1 (1.6 %) | 17 (26.6 %) | 20 (31.3 %) | 23 (35.9 %) | 3.9 (1.1) | 64 |
| I participate in networking events between general practices and hospitals. | 6 (9.4 %) | 10 (15.6 %) | 15 (23.4 %) | 23 (35.9 %) | 10 (15.6 %) | 3.3 (1.2) | 64 |
| I have personal contacts with some GPs in the region/ in the hospital (quality circles, continuing education). | 12 (18.8 %) | 6 (9.4 %) | 13 (20.3 %) | 17 (26.6 %) | 16 (25 %) | 3.3 (1.4) | 64 |

|  | Not at all true | Rather not true | Partly true | Rather true | Very true | mean (SD) | n |
| --- | --- | --- | --- | --- | --- | --- | --- |
| **Resources** |  |  |  |  |  |  |  |
| I consider the remuneration for admission and discharge management measures to be appropriate. | 8 (13.6 %) | 16 (27.1 %) | 22 (37.3 %) | 7 (11.9 %) | 6 (10.2 %) | 2.8 (1.1) | 59 |
| The invoice numbers and modalities in [BLINDED] were easy to understand. | 4 (6.9 %) | 9 (15.5 %) | 13 (22.4 %) | 23 (39.7 %) | 9 (15.5 %) | 3.4 (1.1) | 58 |
| There is sufficient staff available in my hospital/ practice to carry out the [BLINDED] programme. | 11 (18 %) | 30 (49.2 %) | 12 (19.7 %) | 5 (8.2 %) | 3 (4.9 %) | 2.3 (1) | 61 |
| There are sufficient workplaces available in my hospital/ practice to carry out the [BLINDED] programme. | 8 (13.6 %) | 20 (33.9 %) | 20 (33.9 %) | 5 (8.5 %) | 6 (10.2 %) | 2.7 (1.1) | 59 |

|  | Not at all true | Rather not true | Partly true | Rather true | Very true | mean (SD) | n |
| --- | --- | --- | --- | --- | --- | --- | --- |
| **External policy and external requirements** |  |  |  |  |  |  |  |
| The implementation of the requirements of the legal regulation on discharge management complicates the implementation of the [BLINDED] programme. | 4 (8.2 %) | 6 (12.2 %) | 19 (38.8 %) | 16 (32.7 %) | 4 (8.2 %) | 3.2 (1) | 49 |
| The EU GDPR makes cross-institutional and cross-sectoral cooperation more difficult. | 2 (3.7 %) | 5 (9.3 %) | 19 (35.2 %) | 16 (29.6 %) | 12 (22.2 %) | 3.6 (1.1) | 54 |
| The EU GDPR complicates the implementation of the [BLINDED] programme. | 2 (3.7 %) | 7 (13 %) | 16 (29.6 %) | 18 (33.3 %) | 11 (20.2 %) | 3.5 (1.1) | 54 |
| The decision to participate was a strategic one due to [BLINDED]'s high market share. | 5 (10.2 %) | 8 (16.3 %) | 21 (42.9 %) | 12 (24.5 %) | 3 (6.1 %) | 3 (1) | 49 |

|  | Not at all true | Rather not true | Partly true | Rather true | Very true | mean (SD) | n |
| --- | --- | --- | --- | --- | --- | --- | --- |
| **Project management in [BLINDED]** |  |  |  |  |  |  |  |
| I feel that I have fully understood the [BLINDED] project with its goals and contents. | 2 (3.1 %) | 3 (4.7 %) | 10 (15.6 %) | 29 (45.3 %) | 20 (31.3 %) | 4 (1) | 64 |
| The responsibilities of the individual project partners were clearly understood. | 2 (3.2 %) | 4 (6.5 %) | 13 (21 %) | 26 (41.9 %) | 17 (27.4 %) | 3.8 (1) | 62 |
| From the project partners' side, I had enough information and support that I need to implement [BLINDED]. | 2 (3.2 %) | 2 (3.2 %) | 11 (17.5 %) | 31 (49.2 %) | 17 (27 %) | 3.9 (0.9) | 63 |
| I trust one or more institutions involved in the project organisation. | 0 | 2 (3.2 %) | 7 (11.1 %) | 30 (47.6 %) | 24 (38.1 %) | 4.2 (0.8) | 63 |
| From the beginning, I was aware of the fact that [BLINDED] was a study. | 0 | 2 (3.1 %) | 1 (1.6 %) | 13 (20.3 %) | 48 (75 %) | 4.7 (0.7) | 64 |
| From the beginning, I was aware of the fact that the [BLINDED] project has a limited duration. | 0 | 2 (3.1 %) | 1 (1.6 %) | 12 (18.8 %) | 49 (76.6 %) | 4.7 (0.7) | 64 |

|  | Not at all true | Rather not true | Partly true | Rather true | Very true | mean (SD) | n |
| --- | --- | --- | --- | --- | --- | --- | --- |
| **Inclusion of [BLINDED] patients** |  |  |  |  |  |  |  |
| It is difficult to identify patients eligible for participation in [BLINDED]. | 4 (8.2 %) | 14 (28.6 %) | 17 (34.7 %) | 6 (12.2 %) | 8 (16.3 %) | 3 (1.2) | 49 |
| It is difficult to successfully motivate eligible patients to participate in [BLINDED]. | 1 (2.1 %) | 13 (27.7 %) | 19 (40.4 %) | 11 (23.4 %) | 3 (6.4 %) | 3 (0.9) | 47 |
| It is easy for me to communicate the goals and contents of [BLINDED] to eligible patients in an understandable way. | 2 (4.3 %) | 7 (14.9 %) | 17 (36.2 %) | 18 (38.3 %) | 3 (6.4 %) | 3.3 (1) | 47 |
| The [BLINDED] programme meets the needs of my patients. | 1 (2.1 %) | 3 (6.4 %) | 26 (55.3 %) | 15 (31.9 %) | 2 (4.3 %) | 3.3 (0.7) | 47 |
