# Supplementary material for: Hospital Admission and Discharge: Lessons Learned from a Large Programme in Southwest Germany
Source: Int J Integr Care. 2023 Jan 27;23(1):4. doi: 10.5334/ijic.6534 (PMC9881439; doi:10.5334/ijic.6534)
Supplement: TIDieR list, Additional Files 1–10. — Tables on the results of the effectiveness analysis and results of the quantitative survey. [file ijic-23-1-6534-s1.zip › s1-ijic-6534_forstner/6534-24603-1-SP.docx]

Additional File 9

Results of the [BLINDED] programme as perceived by the participants of the survey

|  | Not at all true | Rather not true | Partly true | Rather true | Very true | mean (SD) | n |
| --- | --- | --- | --- | --- | --- | --- | --- |
| Through [BLINDED], contact with patients could be intensified. | 12 (21.9 %) | 4 (7.1 %) | 14 (25 %) | 17 (30.4 %) | 9 (16.1 %) | 3.1 (1.4) | 56 |
| Through [BLINDED], closer contact could be established with the admitting physicians/ discharging hospitals. | 19 (33.9 %) | 12 (21.4 %) | 15 (26.8 %) | 9 (16.1 %) | 1 (1.8 %) | 2.3 (1.2) | 56 |
| Through [BLINDED], VERAHs are better integrated into care provision than before. | 5 (8.9 %) | 6 (10.7 %) | 19 (33.9 %) | 25 (44.6 %) | 1 (1.8 %) | 3.2 (1) | 56 |
| Through [BLINDED], patients could be better involved in decisions regarding further care than before. | 4 (7 %) | 15 (26.3 %) | 24 (42.1 %) | 13 (22.8 %) | 1 (1.8 %) | 2.9 (0.9) | 57 |
| Through [BLINDED], patients had a better understanding of their own care than before. | 5 (9.1 %) | 3 (5.5 %) | 27 (49.1 %) | 19 (34.5) | 1 (1.8 %) | 3.1 (0.9) | 55 |
| Through [BLINDED], patients were made aware of hospital departments participating in [BLINDED]. | 21 (38.2 %) | 11 (20 %) | 9 (16.4 %) | 13 (23.6 %) | 1 (1.8 %) | 2.3 (1.3) | 55 |
| The [BLINDED] feedback meetings have supported networking between hospitals and general practices. | 7 (13.2 %) | 18 (34 %) | 7 (13.2 %) | 18 (34 %) | 3 (5.7 %) | 2.9 (1.2) | 53 |
| Through [BLINDED], to some extent, general practices/ hospitals work together that would not have worked together before. | 24 (43.6 %) | 17 (30.9 %) | 8 (14.5 %) | 4 (7.3 %) | 2 (3.6 %) | 2 (1.1) | 55 |
| Through [BLINDED], there was a better exchange of information between general practice and hospital. | 15 (27.3 %) | 19 (34.5 %) | 6 (10.9 %) | 13 (23.6 %) | 2 (3.6 %) | 2.4 (1.2) | 55 |
| [BLINDED] has improved cooperation across care sectors. | 13 (23.6 %) | 16 (29.1 %) | 11 (20 %) | 13 (23.6 %) | 2 (3.6 %) | 2.6 (1.2) | 55 |
| Through [BLINDED], my awareness was raised that cooperation with other care providers is important. | 9 (16.1 %) | 4 (7.1 %) | 17 (30.4 %) | 21 (37.5 %) | 5 (8.9 %) | 3.1 (1.2) | 56 |
