# Supplementary material for: Hospital Admission and Discharge: Lessons Learned from a Large Programme in Southwest Germany
Source: Int J Integr Care. 2023 Jan 27;23(1):4. doi: 10.5334/ijic.6534 (PMC9881439; doi:10.5334/ijic.6534)
Supplement: TIDieR list, Additional Files 1–10. — Tables on the results of the effectiveness analysis and results of the quantitative survey. [file ijic-23-1-6534-s1.zip › s1-ijic-6534_forstner/6534-24604-1-SP.docx]

Additional File 10

Attractiveness and acceptance of the [BLINDED] programme

|  | Not at all true | Rather not true | Partly true | Rather true | Very true | mean (SD) | n |
| --- | --- | --- | --- | --- | --- | --- | --- |
| I would recommend participation in [BLINDED] to colleagues. | 3 (4.9 %) | 13 (21.3 %) | 19 (31.3 %) | 23 (37.7 %) | 3 (4.9 %) | 3.2 (1) | 61 |
| I would recommend participation in [BLINDED] to all my patients. | 7 (10.9 %) | 8 (12.5 %) | 17 (26.6 %) | 26 (40.6 %) | 6 (9.4 %) | 3.3 (1.1) | 64 |
| I would like to see all general practices participating in [BLINDED]. | 4 (6.7 %) | 7 (11.7 %) | 11 (18.3 %) | 31 (51.7 %) | 7 (11.7 %) | 3.5 (1.1) | 60 |
| I would like to see all hospitals participating in [BLINDED]. | 4 (6.3 %) | 4 (6.3 %) | 9 (14.3 %) | 31 (49.2 %) | 15 (23.8 %) | 3.8 (1.1) | 63 |
| I would like to see all health insurance funds participate in [BLINDED]. | 4 (6.3 %) | 7 (11.1 %) | 11 (17.5 %) | 24 (38.1 %) | 17 (27 %) | 3.7 (1.2) | 63 |
| Participation in [BLINDED] strengthens the role of primary care. | 3 (4.8 %) | 2 (3.2 %) | 14 (22.2 %) | 31 (49.2 %) | 13 (20.6 %) | 3.8 (1) | 63 |
| Participation in [BLINDED] strengthens the role of the VERAH. | 3 (4.9 %) | 2 (3.3 %) | 12 (19.7 %) | 25 (41 %) | 19 (31.1 %) | 3.9 (1) | 61 |
| The conduct of the [BLINDED] programme can be delegated to a large extent to VERAHs/nurses. | 2 (3.2 %) | 4 (6.3 %) | 17 (27 %) | 29 (46 %) | 11 (17.5 %) | 3.7 (0.9) | 63 |
| The conduct of the [BLINDED] programme is too bureaucratic. | 0 | 4 (6.6 %) | 18 (29.5 %) | 22 (36.1 %) | 17 (27.9 %) | 3.9 (0.9) | 61 |
| The conduct of the [BLINDED] programme leads to double documentation. | 1 (1.6 %) | 4 (6.3 %) | 13 (20.6 %) | 26 (41.3 %) | 19 (30.2 %) | 3.9 (1) | 63 |
| The conduct of the [BLINDED] programme can be well integrated into internal processes. | 13 (20.6 %) | 18 (28.6 %) | 24 (38.1 %) | 6 (9.5 %) | 2 (3.2 %) | 2.5 (1) | 63 |
